# Effects of Earthquake Fear and Post-Traumatic Stress Disorder on Patients with Irritable Bowel Syndrome after a Major Earthquake in Türkiye: A Multicenter Study

**DOI:** 10.5152/tjg.2026.25453

**Published:** 2026-01-06

**Authors:** Rasim Eren Cankurtaran, Hulusi Can Karpuzcu, Engin Ataman, Gokhan Aydin, Kenan Kosar, Sedat Cicek, Fatih Kivrakoglu, Batuhan Baspinar, Emre Dirican

**Affiliations:** 1 Department of Gastroenterology, Ankara Etlik City Hospital, Ankara, Türkiye; 2 Department of Gastroenterology, ahramanmaraş Necip Fazıl City Hospital, Kahramanmaraş, Türkiye; 3 Department of Gastroenterology, İskenderun State Hospital, Hatay, Türkiye; 4 Department of Gastroenterology, Adıyaman Training and Research Hospital, Adıyaman, Türkiye; 5 Department of Gastroenterology, Osmaniye State Hospital, Osmaniye, Türkiye; 6 Department of Gastroenterology, Kilis Prof. Dr Alaeddin Yavaşca State Hospital, Kilis, Türkiye; 7 Department of Medical Informatics and Biostatistics, Mustafa Kemal University Faculty of Medicine, Hatay, Türkiye

**Keywords:** Earthquake, fear of earthquake, irritable bowel syndrome, post-traumatic stress disorder

## Abstract

**Background/Aims:**

: On February 6, 2023, 2 devastating earthquakes struck south-eastern Türkiye, causing over 100 000 injuries and more than 50 000 deaths. This study aimed to investigate the impact of post-traumatic stress disorder (PTSD) and fear of earthquake on the severity of irritable bowel syndrome (IBS) symptoms and IBS-related quality of life (IBS-QoL).

**Materials and Methods:**

: Participants diagnosed with IBS were categorized into 2 groups: those residing in earthquake zones and those in non-earthquake zones. Data regarding demographic characteristics, IBS Symptom Severity Scale (IBS-SSS), IBS-QoL, Post-traumatic Stress Disorder Checklist for Diagnostic and Statistical Manual of Mental Disorders-5th edition (PCL-5), and Fear of Earthquake Scale (FES) were collected through validated questionnaires. Multivariate analyses, multiple linear regression, and elastic net (EL) models were performed to identify predictors of IBS-SSS and IBS-QoL scores.

**Results:**

A total of 225 IBS patients were included, with 117 (52%) from earthquake zones and 108 (48%) from non-earthquake zones. Mean IBS-SSS, IBS-QoL, FES, and PCL-5 scores were significantly higher in the earthquake group compared to the non-earthquake group (249 vs. 141; 44.1 vs. 22.8; 20 vs. 9; 47 vs. 28, respectively; *P* < .001 for all). The PCL-5 and FES scores were independent predictors of IBS-SSS (OR = 1.057, *P* < .001 and OR = 1.082, *P* = .019). In regression and EL models, PCL-5 (*P* < .001) was the strongest predictor (100%) and FES (*P* = .006) the second (38%) for IBS-QoL.

**Conclusion:**

The PTSD and earthquake-related fear significantly impact IBS symptom severity and QoL. A holistic treatment approach addressing psychosomatic and mental health factors may improve outcomes in IBS patients.

**Main Points**

- This study aimed to investigate the effects of post-traumatic stress disorder (PTSD) and earthquake-related fear on IBS symptom severity and quality of life (QoL) in patients diagnosed with irritable bowel syndrome (IBS).
- Patients with IBS living in earthquake-affected areas had significantly higher IBS Symptom Severity Scale, IBS-QoL, Post-traumatic Stress Disorder Checklist for Diagnostic and Statistical Manual of Mental Disorders-5th edition (PCL-5), and Fear of Earthquake Scale scores than those living in non-earthquake zones.
- The PTSD severity (PCL-5) and fear of earthquake were found to be independent predictors of both IBS symptom severity and reduced QoL.
- A holistic treatment approach addressing both gastrointestinal and mental health aspects may be beneficial in IBS management.

## Introduction

Irritable bowel syndrome (IBS) is a common functional gastrointestinal disorder characterized by chronic abdominal pain and changes in stool frequency or consistency.^1^ It is the most commonly diagnosed functional gastrointestinal disorder, accounting for approximately one-third of all gastroenterology admissions.^2^ Although there are regional variations, the prevalence of IBS is generally estimated to be between 10% and 15%.^3^^,^^4^ The direct or indirect cost of IBS has been reported to be between $800 and $7700 per patient per year.^5^ It is also known that IBS is a disease that is not limited to treatment costs, but also negatively affects the quality of life (QoL) of patients, causes fatigue, restricts physical activities, and keeps their general health perception lower than the general population.^6^

Although IBS is a common disorder, its pathophysiology has not been clearly elucidated. With ongoing research and the updating of the Rome criteria, the mechanism of brain-bowel interaction has come to the fore in recent years.^7^^,^^8^ Psychosocial factors are one of the important factors in the etiology of IBS.^9^ It has been reported that abuse and trauma in early life are associated with the development of IBS in adulthood.^10^ Post-traumatic stress disorder (PTSD) is an important clinical condition associated with IBS.^11^ The PTSD is characterized by symptoms following exposure to a physical or emotional traumatic event, such as re-experiencing the event, avoidance of triggers, development of negative thoughts and moods, and symptoms of chronic hyperarousal.^12^ Many recent studies have found PTSD to be an independent risk factor for IBS.^13^^,^^14^ In these studies, it was observed that IBS symptoms were common in soldiers returning after war.

On February 6, 2023, 2 major earthquakes measuring 7.7 and 7.8 on the Richter scale struck south-eastern Türkiye, causing over 100 000 injuries and more than 50 000 deaths. In addition, many problems such as shelter, nutrition, and access to health services emerged after the earthquake, and many people had to migrate to other cities.^15^ The earthquake not only caused financial loss and physical damage but also had a significant negative impact on people’s mental health.^16^ A recent study reported that nearly one-fifth of individuals affected by the February 6 earthquake continued to meet the diagnostic criteria for PTSD even at the end of the first year.^17^ Although numerous publications in the literature have investigated various bodily systems in individuals affected by this earthquake, no articles were identified that examined its effects on the gastrointestinal system.^18^^,^^19^ Focusing specifically on IBS among gastrointestinal disorders, it was observed that previous studies investigating the effects of earthquakes on this condition were also highly limited.^20^

This study aimed to evaluate the impact of PTSD and earthquake-related fear on symptom severity and QoL in patients with IBS. An additional aim was to shed light on the effect of the earthquake on patients with IBS by comparing patients with IBS in the earthquake zone with patients in the non-earthquake zone. Furthermore, the study sought to contribute to the limited existing literature on how earthquakes may affect IBS.

## Materials and Methods

### Study Design and Participants

This was a cross-sectional study conducted in 6 centers in Turkiye between February 3, 2024, and March 3, 2024. All participants with IBS were divided into 2 groups: the earthquake zone group and the control group. Five centers from the earthquake-affected region (Hatay, Adıyaman, Kahramanmaraş, Osmaniye, and Kilis) and 1 center from Ankara (as the control site) participated in the study. The study included consecutive adult outpatients diagnosed with IBS according to Rome IV criteria at gastroenterology clinics, regardless of subtype, who gave informed consent. Patients with severe mood disorders, impaired mental function, organ failure, or organic gastrointestinal diseases (e.g., IBD, pancreatitis, or malignancy) were excluded. Individuals who were unable to complete the questionnaire in Turkish and those who refused to participate in the study were also excluded. In addition, patients who had migrated to the control center after experiencing the earthquake were excluded from the control group to avoid misclassification. Ethical approval was obtained from the Ankara Etlik City Hospital ethics committee, and the study was conducted in accordance with the tenets of the Declaration of Helsinki (Date: January 31, 2024, approval number: AEŞH-BADEK-2024-094). Written informed consent was obtained from all participants prior to enrollment in the study

### Data Collection

Data related to demographic characteristics such as age, gender, comorbidities, educational, and marital status were obtained by questionnaires from the participants. Information such as injuries in the earthquake, death of relatives, damage to the house, and economic losses were also obtained from the patients in the earthquake zone group. The IBS Symptom Severity Scale (IBS-SSS) was used for the IBS severity score and the IBS-QOL scale for IBS-related QoL. The PTSD Checklist-5 (PCL-5) scale, developed by summarizing the Diagnostic and Statistical Manual of Mental Disorders-5th edition (DSM-5), was used to measure PTSD symptoms. In order to evaluate the level of earthquake fear, the Fear of Earthquake Scale (FES) was used.

### Irritable Bowel Syndrome Symptom Severity Scale

The IBS-SSS is a summary score that includes several IBS-related measures, such as intensity of abdominal pain, frequency of pain episodes, bloating, satisfaction with bowel habits, and QoL, each scored from 0 to 100. The total score ranges from 0 to 500, providing an overall assessment of IBS severity. Severity classification includes mild (75-175), moderate (175-300), and severe (>300) categories.^21^

### Irritable Bowel Syndrome Quality of Life

According to Patrick et al^22^ and Drossman et al,^23^ a questionnaire consisting of 34 items was administered, each rated on a 5-point Likert scale. The questionnaire covers 8 subscales: dysphoria, interference with activity, body image, health worry, food avoidance, social reactions, sexual issues, and relationships. Scores for each subscale range from 0 to 100. Higher scores on this instrument are indicative of lower QoL. The validated Turkish version of the IBS-QoL questionnaire validated by Uran et al^24^ was used in this study.

### Post-traumatic Stress Disorder Checklist for Diagnostic and Statistical Manual of Mental Disorders—Fifth Edition

The PCL-5, as described by Weathers et al^25^ and revised by Blevins et al,^26^ is a 20-item self-report questionnaire designed to assess PTSD symptoms according to DSM-5 criteria. Each symptom is rated on a 5-point Likert scale ranging from 0 (not at all) to 4 (extremely). The cumulative scores of all items give a total symptom severity score ranging from 0 to 80. The Turkish version of the PCL-5 was validated by Boysan et al,^27^ who found a validated cut-off score of 47 for the purposes of this study.

### Fear of Earthquake Scale

The FES was developed to measure the level of fear associated with earthquakes.^28^ It consists of 7 items and uses a 5-point Likert scale ranging from 1 (“strongly disagree”) to 5 (“strongly agree”). The total score on this scale ranges from 7 to 35 points. The Turkish adaptation of the questionnaire was developed by Sarı et al.^29^

### Statistical Analysis and Simple Size

In this study, the data were analyzed using SPSS 25 (IBM SPSS Corp.; Armonk, NY, USA) and R Studio Team (2020). (RStudio; Boston, MA, USA) http://www.rstudio.com/ 2023.06.1+524 version, “caret v6.0-94,” “dplyr v1.1.4,” and “elastic net v1.3” packages. Mean, standard deviation, median (Q1-Q3), frequency, and percentage values were used for descriptive statistics. Normality assessment was made with Shapiro–Wilk and Kolmogorov–Smirnov tests. Student’s *t*-test, Mann–Whitney *U*, and chi-square tests were used in univariate analyses. Univariate and multivariate logistic regression (with enter method) analyses were performed for the case where IBS-SSS categories (moderate-severe) were the dependent variable. Odds ratio (OR) and 95% CI are given for this analysis. For the case where IBS-QOL scores were the dependent variable, univariate analyses, correlation analysis, and the routine comparison tests mentioned above were used. For the dependent variable in question, linear regression analysis was performed by ensuring model qualifications (linearity, independence, homoscedasticity, normality, and multicollinearity) with the backward elimination method. In addition, an elastic net (EL) model was constructed with the same independent variables for both deviations from simplistic qualifications and variable importance for the dependent variable in question. The EL procedure is a form of regularized optimization for linear regression that provides a bridge between ridge regression and the lasso.^30^ In addition, this method simultaneously does automatic variable selection and continuous shrinkage, and it can select groups of correlated variables.^31^ After the parameters were determined *α* = 0.3, *λ* = 2.36 for final EL model) with 10-fold cross validation (all data were accepted as both training and test sets), the order of importance of the variables contributing to the prediction was determined.

In the general score types (FES, PCL-5, IBS-SSS, IBS-QOL), a pilot study conducted with those from the earthquake zone (n = 20) and those from the non-earthquake zone (n = 20) found high effect sizes and the required sample sizes for each score type (1.72 (n = 14), 0.71 (n = 68), 0.95 (n = 38), 0.65 (n = 176), respectively). Sample sizes were calculated by taking the 2-sided hypothesis into account, assuming the type I error was 0.05 for 80% statistical power. However, due to higher power (99%) and possible data loss, it was decided to conduct the study with a total of 225 individuals. G*Power 3.1.9.4 program was used in statistical power calculations.

## Results

Out of 241 IBS patients, 225 were included in the study, 117 (52%) from earthquake centers and 108 (48%) from non-earthquake centers. Due to incomplete questionnaire responses or refusal to participate, a total of 16 IBS patients were excluded from the study. Age (*P* = .137), sex (*P* = .763), marital status (*P* = .513), educational status (*P* = .289), place of residence (*P* = .137), and other variables showed similar differences between the 2 groups. Significant differences were likely to be found for some of the earthquake-specific questions. The detailed results of the variables are shown in Table 1.

When the scale scores were evaluated according to earthquake experience (Table 2), it was observed that all of the total scale scores were higher in patients in the earthquake zone. Scores for IBS-SSS, IBS-QOL, FES, and PCF-5 were significantly higher in the earthquake region (249, 44.1, 20, and 47, respectively) compared to the non-earthquake region (141, 22.8, 9, and 28, respectively) (*P* < .001 for all).

**Figure 1.**
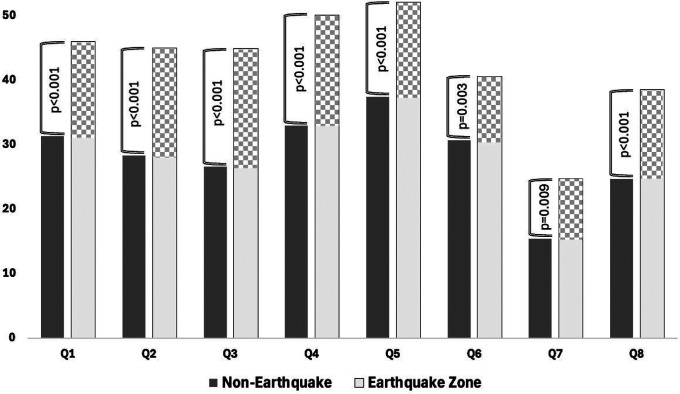
Between-group comparison of IBS-QOL subscale scores (Q1: Dysphoria, Q2: Interference with activity, Q3: Body image, Q4: Health worry, Q5: Food avoidance, Q6: Social reaction, Q7: Sexual activity, Q8: Relationships).

**Figure 2.**
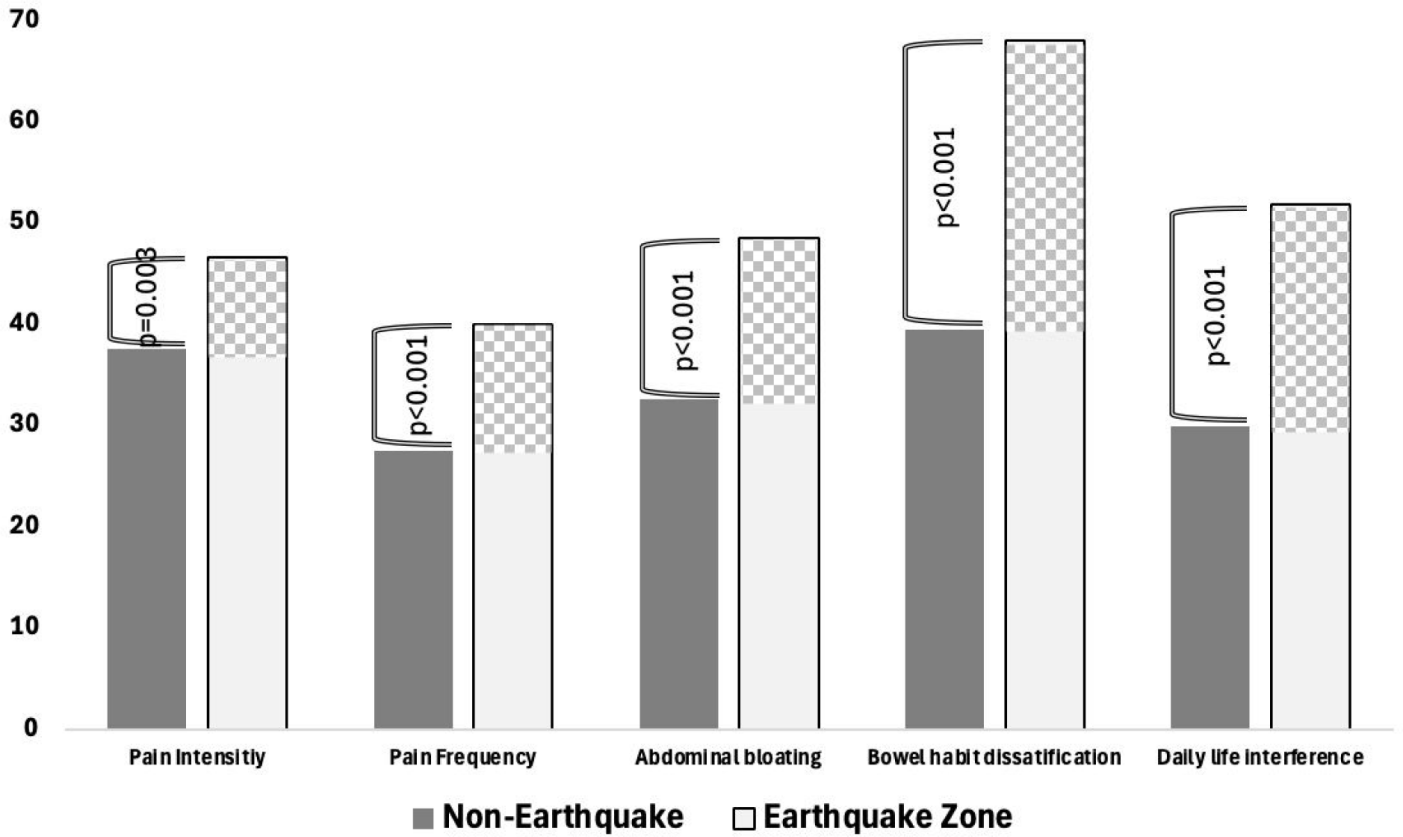
Between-group comparison of IBS-SSS subscale scores.

When IBS-QOL subscores in the Q1-Q8 range were evaluated according to both groups, statistically significant elevation of all subscores in patients in the earthquake zone group was noted (Figure 1). In addition, IBS-SSS subscores such as pain intensity (*P* = .003), pain frequency (*P* < .001), abdominal bloating (*P* < .001), bowel habit dissatisfaction (*P* < .001), and daily life interference (*P* < .001) were also significantly higher in the earthquake zone group (Figure 2).

Independent risk factors affecting the severity of IBS-SSS were evaluated separately according to the presence of moderate-to-severe IBS by univariate and multiple regression analysis (Table 3). A 10-point increase in FES score was associated with approximately a 2.36-fold higher likelihood of moderate-to-severe IBS-SSS (95% CI: 1.37-4.09), whereas a 10-point increase in PCL-5 score corresponded to a nearly 1.70-fold increase in risk (95% CI: 1.37-2.11). When the effect of living in an earthquake zone as the primary variable on the severity of IBS-SSS was analyzed according to univariate and multivariate approaches when the effect of other variables was controlled, living in an earthquake zone was found to be significant in univariate analysis (OR = 4.882, CI: 2.775-8.591, *P* < .001), but not in multivariate analysis (*P* = .316). However, PCL-5 and FES scores were significant in both univariate analysis (OR = 1.076, CI: 1.055-1.097, *P* < .001 and OR = 1.185, CI: 1.130-1.243, *P* < .001) and multivariate analysis (OR = 1.057, CI: 1.034-1.080, *P* < .001 and OR = 1.082, CI: 1.013-1.156, *P* = .019).

Multiple linear and EL models were created by comparing IBS-QOL scores for possible risk factors and then defining them as dependent variables (Table 4). While gender (*P* < .001), marital status (*P* < .042), educational status (*P* = .03), living in the earthquake zone (*P* < .001), history of psychiatric follow-up (*P* = .015), and some other earthquake-related variables were significant in the baseline comparison, only PCL-5 (*P* < .001) and FES (*P* = .006) were significant in the multiple model. In addition, according to the EL model, PCL-5 was the most important variable (100%) and FES was the second most important variable (38%) in predicting IBS-QOL scores.

## Discussion

This study found that IBS patients in the earthquake zone had higher IBS severity and QoL scores than IBS patients in the non-earthquake zone. In addition, the PTSD and FES scores of the patients in the earthquake zone were significantly higher than those in the control group. However, the main important findings of the study were that living in the earthquake zone was not found to be an independent risk factor in terms of IBS-SSS and IBS-QOL, while PCL-5, which is an indicator of PTSD, and FES scores, which are indicators of fear of earthquakes, were found to be independent risk factors for both scores.

Although the pathophysiology of IBS is still not clearly elucidated today, there are increasing views on the mechanism of the brain-gut axis.^32^ Many studies have put forward various hypotheses in this direction.^33^^,^^34^ In a recent review, it was emphasized that the gut microbiota is effective in the gut-brain axis interaction, and for this reason, IBS is associated with many neuropsychiatric disorders.^34^

Post-traumatic stress disorder is one of the important clinical conditions whose relationship with IBS has been investigated in recent years. Irritable bowel syndrome is highly associated with other neuropsychiatric disorders and PTSD. In a study investigating the association of neuropsychiatric disorders with IBS, it was reported that 54% of IBS patients had a history of psychiatric illness, 44% had a history of trauma, and 36% had current PTSD.^35^ Recent studies have suggested that there are both molecular and genetic factors that may explain the association between IBS and PTSD.^36^^,^^37^ In particular, neuropeptide Y levels and activity have been reported to play a key role in the association between PTSD and IBS.^37^ Additionally, several hypotheses have proposed that this relationship may be mediated through dysregulation of the hypothalamic–pituitary–adrenal axis, alterations in gut–brain axis signaling, and stress-related changes in the intestinal microbiota.^38^ Although the underlying mechanism is still unclear, the close relationship and association between PTSD and IBS has been the subject of research in many studies. In a meta-analysis of 8 studies, the presence of PTSD was associated with an increased likelihood of IBS.^9^ Another study reported that PTSD in veterans was a significant risk factor for IBS in a condition called Gulf War illness, which is characterized by some gastrointestinal symptoms.^39^ In another study including veterans with symptomatic PTSD, IBS was found to be more common than in the general population, and severe PTSD was found to be a risk factor for the severity of gastrointestinal symptoms such as diarrhea, constipation, and bloating.^40^ In the study, PTSD severity was a significant risk factor for IBS-SSS, which is similar to this study. In a recent population-based study involving 1617 civilians exposed to war, it was found that some war-related events were significantly associated with IBS. Another important result of this study was that a significant association was found between PTSD and IBS.^41^ The study was related to earthquake and IBS, and the population without earthquake exposure was also included in the study. No association was identified between earthquake-related factors such as death of a family member, financial problems, trauma, and IBS severity in the study. However, although these data related to earthquake exposure were included in the study, making a comparison with this study may not be appropriate due to the lack of a standardized trauma exposure scale, the inclusion of the population without exposure, and the lower number of participants in the study compared to population studies.

Until this part of the article, all the studies examining the relationship between PTSD and IBS in the literature consisted of veterans and civilians exposed to war.^14^^,^^39^^-^^41^ Another important objective of the study was to evaluate the effect of earthquake fear on IBS severity and QoL. However, only a limited number of studies investigating the relationship between earthquake and IBS were identified in the literature.^20^ In that study, it was reported that the implementation of a health education program for students diagnosed with IBS after the earthquake significantly decreased IBS-QOL scores and had a positive effect on the participants. Although that was an interventional study and therefore not directly comparable with the cross-sectional design, it further reinforces the notion that earthquake-related psychosocial distress can meaningfully influence IBS outcomes. In line with those findings, the results similarly indicate that post-traumatic psychological factors such as PTSD and earthquake-related fear may play a critical role in symptom burden and QoL among IBS patients. In the study, the FES score, which is an earthquake fear scale, was an independent risk factor for IBS-SSS and IBS-QOL, just like PTSD. The fact that FES also significantly increased the severity of IBS shows us that psychosocial factors should be evaluated as a triggering factor. Although the IBS-SSS and IBS-QOL scores of IBS patients in the earthquake zone were higher than those in the non-earthquake zone, living in the earthquake zone was not an independent risk factor affecting these scores. In this case, the presence of patients with severe IBS in the non-earthquake region and the presence of patients with high PTSD scores due to other causes, such as abuse and trauma, may be effective.

The study had several limitations. The first was that the study started 1 year after the earthquake. This may have influenced and weakened the association between IBS and the earthquake. Another limitation was that the study was not a population-based field survey, but a cross-sectional study of patients who presented to the participating health centers, without longitudinal follow-up. Finally, although the prevalence of psychiatric history or current psychiatric follow-up was included in the study, detailed information on the use of psychotropic medications was not available. This may be considered a limitation, as such medications could potentially influence IBS symptoms.

In conclusion, this study found that PTSD and FES scores were independent risk factors affecting IBS severity and IBS-related QoL. While evaluating these patients, it should be kept in mind that underlying psychosocial factors may also be effective in the disease. Patients with IBS may benefit from a holistic approach to treatment, including mental health and psychosomatic conditions.

## Figures and Tables

**Table 1.** Baseline and Demographic Characteristics of Participants

Variables	Total (n = 225)	Non-Earthquake (n = 108)	Earthquake Zone (n = 117)	*P*
Age	37.1 ± 12.1	38.8 ± 13.3	36.4 ± 10.8	.137
Female gender	146 (64.9)	69 (63.9)	77 (65.8)	.763
Marital status				
Single/Widow	82 (36.4)	37 (34.3)	45 (38.5)	.513
Married	143 (63.6)	71 (65.7)	72 (61.5)	
Education status				
Primary school	56 (24.9)	25 (23.1)	31 (26.5)	.289
High school	58 (25.8)	33 (30.6)	25 (21.4)	
University	111 (49.3)	50 (46.3)	61 (52.1)	
Living place				
City center	143 (63.6)	74 (68.5)	69 (59)	.137
County	82 (36.4)	34 (31.5)	48 (41)	
Current smoker	78 (34.7)	42 (38.9)	36 (30.8)	.201
Alcohol consumption	32 (14.2)	19 (17.6)	13 (11.1)	.164
Prescription or illicit substance abuse	4 (1.8)	4 (3.7)	0 (0)	.052
History of psychiatric follow-up	105 (46.7)	55 (50.9)	50 (42.7)	.219
Colonoscopy in past 5 years	76 (33.8)	46 (42.6)	30 (25.6)	**.007**
Injury in earthquake	5 (2.2)	0 (0)	5 (4.3)	.061
Death of first-degree relatives in earthquake	7 (3.1)	0 (0)	7 (6)	**.015**
Having an unusable house in earthquake	38 (16.9)	0 (0)	38 (32.5)	**<.001**
Earthquake-related financial problems	45 (20)	0 (0)	45 (38.5)	**<.001**

Continuous variables are presented as mean standard deviation and categorical variables as n (%). Bold values indicate statistical significance (p < 0.05).

**Table 2.** Comparison of Scale Scores According to Earthquake Experience

Variables	Non-Earthquake	Earthquake Zone	*P*
IBS-SSS (total score)	141 (95.5-216)	249 (174-307)	**<.001**
IBS-QOL	22.8 (14-41.2)	44.1 (32.4-57.4)	**<.001**
FES	9 (7-12)	20 (16-27)	**<.001**
PCL-5	28 (14-47.5)	47 (31-56)	**<.001 **

Variables are described by median (Q1-Q3). Bold values indicate statistical significance (p < 0.05).

FES, Fear of Earthquake Scale; IBS-QOL, irritable bowel syndrome-related quality of life; IBS-SSS, Irritable Bowel Syndrome Symptom Severity Scale; PCL-5, Posttraumatic Stress Disorder Checklist for DSM-5 Post-traumatic Stress Disorder Checklist for DSM-5.

**Table 3.** Analysis of the Effect of Variables on Moderate-to-Severe IBS-SSS

Variables	Univariate Analysis		Multivariate Analysis	
	OR (95% Cl)	*P*	OR (95% Cl)	*P*
Age	0.993 (0.972-1.015)	.551		
Female gender	0.426 (0.243-0.745)	.003	0.877 (0.207-1.726)	.58
Marital status				
Single/Widow				
Married	1.4 (0.812-2.414)	.226		
Education status				
Primary school				
High school	0.519 (0.245-1.099)	.087		
University	0.546 (0.282-1.057)	.073		
Living place				
City centre				
County	1.050 (0.609-1.809)	.861		
Living in an earthquake zone				
No				
Yes	4.882 (2.775-8.591)	**<.001**	1.630 (0.627-4.235)	.316
History of psychiatric follow-up	0.675 (0.398-1.144)	.144		
Injury in earthquake	3.652 (0.402-33.2)	.25		
Death of first-degree relatives in earthquake	5.575 (0.660-47.084)	.114		
Having an unusable house in earthquake	3.473 (1.559-7.737)	**.002**	1.074 (0.358-3.224)	.898
Earthquake-related financial problems	4.675 (2.128-10.270)	**<.001**	1.484 (0.528-4.165)	.454
PCL-5	1.076 (1.055-1.097)	**<.001**	1.057 (1.034-1.080)	**<.001**
FES	1.185 (1.130-1.243)	**<.001**	1.082 (1.013-1.156)	**.019**

According to the significant results in the univariate model, a multivariate model was constructed.

FES, Fear of Earthquake Scale; PCL-5, Post-traumatic Stress Disorder Checklist for DSM-5.

**Table 4.** Univariate Analyses and Model Predicting IBS-QOL

Variables	Univariate Analysis		Multivariate Analysis		
			Backward Elimination, VIF = 1.430, 1420 respectively, D.W = 1.879		
IBS-QOL	*P*	Unstandardized Coefficients	Variable Importance, %	*P*
Age	0.046	.491			
FES	0.505	<.001	0.493	38	.006
PCL-5	0.644	<.001	0.58	100	<.001
Gender				0.00	
Female	41.5 (24.3-54.4)	<.001			
Male	22.8 (12.5-44.1)				
Marital status				1.30	
Single/Widow	30.5 (16.2-48.5)	.042			
Married	41.2 (21.3-53.7)				
Education status				0.00	
Primary school	45.6 (17.3-64)	.03			
High school	27.6 (14-44.1)				
University	38.2 (20.6-52.2)				
Living place					
City center	36 (16.9-50.7)	.286			
County	40.8 (20.6-53.7)				
Group				0.00	
Non-zones	22.8 (14-41.2)	<.001			
Earthquake zone	44.1 (32.4-57.4)				
History of psychiatric follow-up				0.00	
Absent	32.4 (14.3-49.3)	.015			
Present	39.7 (22.8-54.4)				
Injury in earthquake					
Absent	36 (16.9-52.6)	.5			
Present	44.1 (41.9-47.1)				
Death of first-degree relatives in earthquake				0.00	
Absent	36 (16.9-52.2)	.267			
Present	46.3 (44.1-47.8)				
Having an unusable house in earthquake				0.00	
Absent	32.4 (16.9-52.2)	.009			
Present	44.5 (38.2-52.9)				
Earthquake-related financial problems				0.00	
Absent	30.2 (16.2-51.5)	<.001			
Present	44.1 (36-58.8)				

IBS-QOL median (Q1-Q3) values of categorical variables were compared with the Mann–Whitney *U*-test. Pearson correlation values between FES, PCL-5, and IBS-QOL were calculated. Multiple linear regression and elastic net models were established in multivariate analyses. Variable importance expresses the variable importance relative to the elastic net.

D.W: Durbin–Watson account value; IBS-QOL, irritable bowel syndrome-related quality of life; VIF, variance inflation factor.

## Data Availability

The data that support the findings of this study are available on request from the corresponding author.
